# Role of Renin–Angiotensin-converting Enzyme Level and ACE Gene Polymorphism in Patients with Nonalcoholic Fatty Liver Disease

**DOI:** 10.5005/jp-journals-10018-1186

**Published:** 2016-12-01

**Authors:** Demet D Tekatas, Ibrahim H Bahcecioglu, Murat Ispiroglu, Abdurrahman Sahin, Necip Ilhan, Mehmet Yalniz, Ulvi Demirel

**Affiliations:** 1 Department of Gastroenterology, Firat University, Elazig, Turkey; 2 Department of Biochemistry, Firat University, Elazig, Turkey

**Keywords:** Angiotensin-converting enzyme, Angiotensin-converting enzyme gene polymorphism, Nonalcoholic fatty liver disease.

## Abstract

**Introduction:**

In this study, we aimed to investigate the histological and clinical effect of angiotensin-converting enzyme (ACE) and ACE gene polymorphism in nonalcoholic fatty liver disease (NAFLD) and their roles in the progression of the disease.

**Materials and methods:**

Liver function tests, body mass index, waist circumference, lipid parameters, fasting blood glucose (FBG), hemoglobin A1c (HbA1c), homeostasis model assessment-IR (HOMA-IR), ACE, and ACE gene polymorphism were evaluated in the NAFLD group and control group. The study group was evaluated by dividing the group into four subgroups by ACE gene polymorphism (D/D homozygous, I/I homozygous, D/I heterozygous, I/D heterozygous). Liver biopsies were evaluated according to Brunt Classification.

**Results:**

A total of 31 patients who were diagnosed with NAFLD and 40 healthy individuals were included in the study. The ACE level was found to be 11.69 ± 1.99 in the NAFLD group and 11.52 ± 1.72 in the control group (p = 0.70). There was a negative correlation between ACE levels and HOMA-IR levels (p = 0.008, r= −0.512). Biochemical parameters were not different among ACE gene polimorphism subgroups, except FBG (between D/D, I/D and D/I, I/D; p = 0.02). When the ACE levels were compared in terms of grade and stage, no significant difference was found (for stage and grade p = 0.68). The ACE gene polymorphism subgroups did not differ by histopathologic findings; grade and stage (for grade p = 0.42, for stage p = 0.92).

**Conclusion:**

In this study, we could not find a correlation of ACE and ACE gene polymorphism with metabolic risk factors and the disease severity in NAFLD.

**How to cite this article:**

Tekatas DD, Bahcecioglu IH, Ispiroglu M, Sahin A, Ilhan N, Yalniz M, Demirel U. Role of Renin–Angiotensin-converting Enzyme Level and ACE Gene Polymorphism in Patients with Nonalcoholic Fatty Liver Disease. Euroasian J Hepato-Gastroenterol 2016;6(2):137-142.

## INTRODUCTION

Nonalcoholic fatty liver disease (NAFLD) is a hepatic disease that may show a clinical course ranging from simple steatosis to fibrosis and hepatic cirrhosis.^1,2^ Primarily, type 2 diabetes mellitus (DM), obesity, and hyperlipidemia and secondarily drugs, bypass surgery, pregnancy, lipid metabolism diseases, and total parenteral nutrition are involved in the etiology.^3,4^ It is known that various hormones (leptin, resistin, adiponectin), neurotransmitters (noradrenalin, angiotensin [AT]-II, plasminogen activator inhibitor-1), and proinflammatory cytokines (tumor necrosis factor alpha and interleukin-6) are released from the adipose tissue and that they have fibrinogenic property.^5,6^

Angiotensin-converting enzyme (ACE) is the key enzyme, i.e., involved in transforming AT-I to the potent vasoconstrictor AT-II.^7^ Angiotensin-II is one of the very potent fibrinogenic molecules that encourage extracellular matrix deposition, myofibroblast proliferation and contraction, and inflammatory cytokine release.^8,9^ The ACE gene is located on chromosome 17q23. A series of locus encoding ACE genes with different phenotypical and functional properties has been defined.^8,10^ Angiotensin-converting enzyme gene polymorphism that has been studied most extensively and that corresponds to 50% of the ACE variants in the tissue and plasma is insertion/deletion (I/D) in intron 16 and depends on the presence or absence of 287 base pair (bp) Alu sequence.^11,12^ A 287 base deletion polymorphism in the 16th intron of the ACE gene increases the amount of ACE in the circulation. The effect of ACE gene polymorphism on fibrogenesis has been investigated in different liver diseases and different results have been obtained.

Knowledge of the ACE level and ACE gene polymorphism in patients with NAFLD/nonalcoholic steatohepatitis (NASH) may be beneficial in terms of determining the treatment methods and in the follow-up of the disease progression. In this study, we investigated the ACE level and ACE gene polymorphism in patients with NAFLD/NASH.

## MATERIALS AND METHODS

Female and male patients above the age of 18 who presented to Firat University Hospital, Internal Medicine Gastroenterology Outpatient clinic and diagnosed with NAFLD by liver biopsy were included in the study. Adult (>18 years) patients found to have steatosis on abdominal ultrasonography and a diagnosis of steatosis made by liver biopsy after exclusion of the other liver diseases (viral hepatitis, autoimmune hepatitis, glucose metabolism disorders, hemochromatosis, Alpha-1 antitrypsin deficiency) were recruited to the study. Individuals who accepted to participate in the study after reading the informed consent form approved by the ethics committee were enrolled in this study. Exclusion criteria were concurrent alcohol consumption, pregnancy, systemic diseases, such as cardiovascular disease, renal failure, cerebrovascular disease, severe coronary artery disease, uncontrolled hypertension (HT), malignancy and receiving oncologic treatment, major operation in the last 2 months, patients receiving parenteral nutrition and patients who used drugs which might be hepatotoxic. In addition, patients who daily consumed significantly excessive alcohol for longer than 1 year at any time in the lifetime (>20 gm/day for women, >30 gm/day for men) were not included in the study.

A total of 71 individuals including 31 NAFLD patients (group I) and 40 healthy controls (group II: age- and gender-matched compared with the NAFLD group) were included in the study. Liver biopsies of NAFLD patients were evaluated according to Brunt Classification.

The waist circumference, height, and weight were calculated in all patients and the body mass indexes (BMIs) were calculated. Fasting blood glucose (FBG), total protein, albumin, total bilirubin, direct bilirubin, alanine aminotransferase (ALT), aspartate aminotransferase (AST), alkaline phosphatase (ALP), gamma glutamyl transpeptidase (GGT), prothrombin time (PT), total cholesterol, low-density lipoprotein (LDL) cholesterol, high-density lipoprotein (HDL) cholesterol, very low-density lipoprotein (VLDL) cholesterol, and triglyceride (TG) levels were studied in the serum. Serologically, hepatitis B surface antigen (HBsAg), anti-HBs, hepatitis B e antigen (HBeAg), anti-HBe, anti-hepatitis B core antigen (HbcAg), and anti-HCV were studied and anti-delta immunoglobulin G was studied by the Macro Elisa method.

Fasting blood glucose and insulin levels after a 12-hour fasting period were recorded. The Homeostasis Model Assessment-IR (HOMA-IR) method was used to evaluate insulin resistance. Homeostasis model assessment-IR was calculated using the following formula: (Fasting insulin × FBGmmol/dL)/22.5. A HOMA-IR index above 2.5 was considered insulin resistance.

Liver biopsies of NAFLD patients were evaluated according to Brunt Classification by an expert pathologist.

The ACE levels were studied using BOSTER Immunoleader (EK 0557, Wuhan Boster Biological Technology Co. Ltd, China) ACE kit which operates with enzyme immunoassay method. A 100 base pair deoxyribonucleic acid (DNA) ladder was cultured in 3% agarose gel which was prepared with 1X TBE and in which ethidium-bromide was added in order to specify ACE I/D polymorphism (by staining PZR products). Deoxyribonucleic acids were visualized by ultraviolet transilluminator following electrophoresis at a 100 V 75 mA current. The individuals who were heterozygous for ACE I/D polymorphism were observed as two different bands at 190 and 490 bp regions, whereas the individuals who were homozygous (I/I) were observed at the 490 band and the individuals who were homozygous (D/D) were observed at the 190 band.^13^

### Statistical Analysis

Statistical analysis was performed using Statistical Package for the Social Sciences (SPSS) 12.0 (Inc, version 12.0, Chicago, IL, USA) package program. The distribution properties of the data were determined parametrically and nonparametically using the normalization test. The Student *t*-test or Mann–Whitney U test was used for comparison of the continuous variables of paired groups according to distribution properties. One-way analysis of variance test was used in assessment of more than two independent groups. Subgroup analyses were performed by *post hoc* tests. Crosstabs or cross-tables were used. Pearson correlation test was used to determine the level (degree-severity-stregnth) and direction of the relation between two variables.

## RESULTS

The NAFLD and control groups were similar in terms of age and gender. The demographic and clinical properties of the NAFLD and control groups are shown in Table 1. In the NAFLD group, AST, ALT, ALP, GGT, total cholesterol, LDL cholesterol, VLDL cholesterol, TG, ferritin, FBG, HbA1c, insulin, and HOMA-IR levels were found to be significantly higher compared with the control group. The mean HOMA-IR value was found to be 2.7 ± 3.4 (n = 26) in the NAFLD group and 0.8 ± 0.7 (n = 36) in the control group; the difference was significant (p = 0.002). Comparison of the biochemical parameters in the NAFLD and healthy control groups is shown in Table 2.

**Table 1:** **Table 1:** Demographic and clinical properties of the NAFLD patients and control group

				*NAFLD (n = 31)*		*Control (n = 40)*		*p-value*	
Gender (M/F)				21/10		20/20		>0.05	
Mean age (years) #				34.3 ± 6.5		33.27 ± 6.8		>0.05	
Body weight (kg) #				84.2 ± 13.3		64.6 ± 10.4		<0.001*	
Waist circumference (cm) #				99.3 ± 12.2		83.2 ± 9.8		<0.001*	
Height (cm) #				170.9 ± 9.6		169.2 ± 8.2		>0.05	
BMI #				28.8 ± 4.4		22.4 ± 2.8		<0.001*	
DM		Yes		4		0		<0.01*	
		No		27		40			
HT		Yes		4		0		<0.01*	
		No		27		40			

*p ≤ 0.05, # mean ± standard deviation

**Table 2:** **Table 2:** Comparison of the biochemical parameters of the NAFLD and control groups

		*NAFLD*		*Control Group*		*p-value*	
AST (U/L)		49.35 ± 23.37		24.50 ± 23.58		<0.001*	
ALT (U/L)		73.45 ± 40.41		18.27 ± 8.31		<0.001*	
ALP (U/L)		88.77 ± 41.89		64.55 ± 18.44		0.002*	
GGT (U/L)		97.58 ± 119.88		18.53 ± 8.78		<0.001*	
T.Bilirubin(mg/dL)		0.79 ± 0.54		0.58 ± 0.35		0.53	
PT (sn)		11.91 ± 0.79		11.38 ± 1.98		0.16	
T.protein (g/dL)		7.47 ± 1.39		7.43 ± 0.42		0.85	
Albumin (g/dL)		4.51 ± 0.34		4.52 ± 0.26		0.89	
Ferritin (ng/mL)		159.92 ± 124.3 (n = 25)		86.80 ± 94.58 (n = 18)		0. 043	
Total cholesterol (mg/dL)		217.00 ± 33.66 (n = 30)		164.8 ± 24.47		<0.001*	
LDL cholesterol (mg/dL)		138.36 ± 25.94 (n = 30)		101.87 ± 16.01		<0.001*	
HDL cholesterol (mg/dL)		50.56 ± 11.11 (n = 30)		55.15 ± 11.96		0.10	
VLDL cholesterol (mg/dL)		34.31 ± 23.69 (n = 30)		17.55 ± 7.78		<0.001*	
TG (mg/dL)		171.90 ± 118.22 (30)		88.32 ± 38.99		<0.001*	
HbA1c (%)		5.62 ± 1.67 (n = 23)		4.61 ± 0.34 (n = 35)		0.001*	
Fasting glucose (mg/dL)		94.16 ± 30.87 (n = 30)		71.82 ± 11.74 (n = 39)		<0.001*	
C reactive protein (mg/L)		3.81 ± 1.52 (n = 24)		3.71 ± 2.09		>0.05	
HOMA-IR		2.7 ± 3.4 (n = 26)		0.8 ± 0.7 (n = 36)		0.002*	
Insulin (µIU/mL)		10.60 ± 9.03 (n = 26)		5.26 ± 4.63 (n = 37)		0.03	
ACE (ng/mL)		11.69 ± 1.99		11.52 ± 1.72		0.70	

*p ≤ 0.05

The ACE level was found to be 11.69 ± 1.99 ng/mL in the NAFLD group and 11.52 ± 1.72 ng/mL in the control group (p = 0.70). The ACE level was found to be 11.56 ± 1.79 ng/mL in men and 11.64 ± 1.95 ng/mL in women; the difference was not statistically significant (p = 0.87). When the ACE levels of the patients with and without HT, DM, hyperlipidemia, and hypertriglyceridemia were compared in the NAFLD group, the ACE level was found to be 11.94 ± 1.90 ng/mL in the subjects without HT (n = 27), 12.01 ± 1.96 ng/mL in the subjects with HT (n = 4), 10.18 ± 2.21 ng/mL in the subjects with DM (n = 4), 11.91 ± 1.90 ng/mL in the subjects without DM (n = 27). No statistically significant difference was found in terms of ACE levels regardless of having hyperlipidemia and hypertriglyceridemia (p > 0.05). Although the relationship between ACE level and the AST/ALT, ALT, CRP, BMI was not found, ACE level was negatively correlated with HOMA-IR levels in the NAFLD group(p = 0.08, r = −0.512) (Table 3).

**Table 3:** **Table 3:** Comparison of ACE levels with some biochemical parameters in the NAFLD group

*Correlation*		*r-value*		*p-value*	
ACE–ALT		–0.128		0.49	
ACE–C-reactive protein		–0.106		0.62	
ACE–BMI		–0.202		0.27	
ACE–HOMA-IR		–0.512		0.008*	
ACE–AST/ALT		0.117		0.52	

*p < 0.05

According to the ACE gene polymorphism subgroups, no difference was found in terms of gender (p = 0.64 for the NAFLD group, p = 0.34 for the control group). In the NAFLD group, 10 men (47.6%) and 3 women (30%) carried D/D homozygous genotype, 7 men (33.3%) and 3 women (30%) carried I/I homozygous genotype, 1 man (4.8%) and 3 women (30%) carried D/I heterozygous genotype, and 3 men (14.3%) and 3 women (30%) carried I/D heterozygous genotype. In the control group, D/D homozygous genotype was found in 13 men (65%) and 10 women (50%). Two men (10%) and 5 women (25%) carried I/I homozygous genotype, 2 men (10%) and 4 women (20%) carried D/I heterozygous genotype, and 3 men (15%) and 1 woman (5%) carried I/D heterozygous genotype.

The mean ages of the subgroups in both groups were 33 ± 5.92 years in D/D genotype, 35 ± 7.95 years in I/I genotype, 30 ± 5.64 years in D/I genotype, and 36 ± 6.04 years in I/D genotype (p = 0.15). When the biochemical parameters were evaluated in terms of ACE gene polymorphism, no significant difference was found. When the subgroups were compared between each other, a significant difference was found between the subgroup that carried D/I heterozygous genotype and the subgroup that carried I/D heterozygous genotype (p = 0.03). Again, the FBG was found to be significantly higher in the I/D subgroup (FBG: 97.55 ± 47.46 mg/dL) compared with the D/D subgroup (FBG: 78.1 ± 19.74, p 0.02 mg/dL) and D/I subgroup (FBG: 72.25 ± 15.86 mg/dL, p 0.02 mg/dL). No difference was found when the subgroups were compared between each other in terms of other biochemical parameters.

The patients in the NAFLD group were divided as grade 1 (mild steatosis) and grades 2 to 3 (moderate to severe steatosis). These patients were grouped as stage 1 (initial stage) and stages 2 to 3 (moderate to advanced stage) in terms of stage. Since we did not have grade 3 and stage 4 patients, the patients were divided into two subgroup in terms of grade (grades 1 and 2), and also into two subgroup in terms of stage (stages 1 to 3). According to the stage, the ACE level was found to be 11.74 ± 1.71 ng/mL in stage 1 and 11.5 ± 2.53 ng/mL in stages 2 to 3; the difference was not statistically significant (p = 0.68). When we examined the ACE levels in terms of grade, the ACE level was found to be 11.84 ± 1.26 ng/mL in grade 1 and 11.55 ± 2.53 ng/mL in grade 2. No significant difference was found in terms of grade (p = 0.68). The distribution of the patients according to the ACE gene polymorphism in the NAFLD group by grade and stage is summarized in Tables 4 and 5. No statistically significant difference was found when the NAFLD group was compared in terms of ACE gene polymorphism by grade (p = 0.42; Table 4) and by stage (p = 0.92; Table 5).

**Table 4:** **Table 4:** Comparison of ACE gene polymorphism by grade in NAFLD

		*Grade*					
		*Grade 1, n (%)*		*Grade 2, n (%)*		*Total (n)*	
D/D		6 (40%)		7 (43.8%)		13	
I/I		5 (33.3%)		5 (31.3%)		10	
D/I		2 (13.3%)		0 (0%)		2	
I/D		2 (13.3%)		4 (25%)		6	

**Table 5:** **Table 5:** Comparison of ACE gene polymorphism by stage in NAFLD

		*Stage*					
		*Stage 1, n(%)*		*Stages 2–3, n(%)*		*Total (n)*	
D/D		6 (42%)		7 (41.2%)		13	
I/I		5 (35.7%)		5 (29.4%)		10	
D/I		2 (7.1%)		0 (5.9%)		2	
I/D		2 (14.3%)		4 (23.5%)		6	

## DISCUSSION

In the NAFLD/NASH patients, the frequency of metabolic syndrome is increased and the role of insulin resistance and inflammatory factors has been demonstrated in many studies.^3^ It has been reported that the D/D allele of the ACE gene shows an increase in serum in insulin resistance and related pathologies.^14^

When the ACE gene polymorphism was evaluated, the ACE genotypes were as follows: D/D (41.9%), I/I (32.2%), D/I (6.4%), I/D (19.3%) in the NAFLD group and D/D (57.5%), I/I (7.5%), D/I (15%), I/D (10%) in the control group.

In a study conducted recently, Nagashio et al^15^ proposed that ACE directly affected pancreatic fibrosis in experimental animals, and ACE inhibitors decreased pancreatic fibrosis in animal models. In the study of Oruc et al^16^ conducted with 52 patients with primary biliary cirrhosis (PBC) and 98 controls, the frequency of the D allele was found to be 54% in the PBC patients and 55% in the control group. No correlation was found between ACE genotype and clinical findings, and no difference was found between ACE genotypes and allele frequency. These results showed that local RAS was a significant factor in chronic liver diseases despite systemic RAS. Again, in the study of Oruc et al,^17^ which was conducted with 51 patients with familial pancreatitis, 104 patients with sporadic chronic pancreatitis and 163 controls, patients with different genotypes were compared and it was concluded that ACE gene polymorphism did not contribute significantly to the pathogenesis of chronic pancreatitis and its progression. In the study of Saibeni et al,^18^ which was conducted with 232 patients with inflammatory bowel diseases (IBD) (124 ulcerative colitis, 108 Cohn’s disease) and 99 controls, it was shown that ACE I/D polymorphism was not related with IBD, but the frequency of extrarenal involvement was increased in D allele carriers with ulcerative colitis.

Conclusively, the ACE I/D gene polymorphism which was thought to be related with fibrosis has shown controversial results in different disease groups which involve fibrosis at the base. In our study, we investigated whether the ACE level and ACE gene polymorphism had any effect in NAFLD on histological and clinical progression. We could not find any significant difference in the NAFLD group and control group in terms of ACE levels and histopathological evaluation.

The frequency of obesity has been reported to be 30 to 90% in NAFLD.^13,19^ However, the actually important question is that, is ACE gene polymorphism related with obesity and is it a significant marker in terms of progression to fibrosis. In the study conducted by Fabris et al,^20^ it was observed that the BMI was higher in D allele carriers and abdominal obesity accompanied in men, when the relation of ACE polymorphism with BMI was examined. In I/I genotype carrier women, the BMI was proposed to be low. When we compared the ACE level and BMI levels in the NAFLD group with this objective, we could not find a significant difference. The NAFLD patients were divided into groups in terms of stage, fibrosis, and the degree of the steatosis; we could find no significant difference between the D/D, I/I, D/I, I/D subgroups in terms of ACE gene polymorphism, BMI, and waist circumference.

There are many studies which suggest that there is a strong relation between NAFLD and DM. Diabetes mellitus is present in approximately 30% of the patients with NAFLD. In diabetic patients, the frequency of NAFLD is increased by 2.6 fold.^21,22^ In addition, there are studies suggesting that DM is a strong independent indicator for fibrosis in NAFLD and most patients with advanced fibrosis are diabetic.^23^ For example, in the study of Haukeland et al^24^ which included 88 patients, it was concluded that abnormal glucose tolerance could predict fibrosis independently. When the frequency of DM and mean FBG levels was compared by ACE gene polymorphism in the NAFLD and control groups, the FBG was found to be 78.1 ± 19.74 mg/dL in the D/D subgroup and 97.55 ± 47.46 mg/dL in the I/D subgroup; a significant difference was found between the two groups (p = 0.26). There was also a difference between the D/I subgroup and I/D subgroup (p = 0.28). When all subgroups were compared between each other, no significant difference could be found in terms of DM and FBG. This result may be interpreted such that DM and FBG do not affect the degree of fibrosis and steatosis by ACE gene polymorphism.

Studies have shown that the insulin resistance in NAFLD is increased compared with the normal population. In our study, a correlation between the ACE level and HOMA-IR was present, but no correlation was found between the ACE level and BMI. However, there was no significant difference in HOMA-IR correlation in the ACE subgroups. Thus, we concluded that HOMA-IR was increased in the NAFLD group as an indicator of insulin resistance, the ACE levels were also increased, and so, it was a significant factor in terms of adiposity and progression to fibrosis. But insulin resistance was not a significant factor in the subgroups in terms of ACE gene polymorphism.

Nonalcoholic fatty liver disease is observed with a 5- to 6-fold higher rate in patients with hyperlipidemia compared with the normal population.^25^ When we compared the two groups in terms of total cholesterol, LDL, VLDL, HDL, TG, a statistically significant difference was found, but no difference was found between the ACE subgroups. We cannot state that the increased AST and ALT levels in NAFLD compared with the control group might be related with the ACE level, since the ACE levels were significantly different in the two groups. In the study conducted by Fabris et al,^20^ it was found that ACE subgroups and increased BMI were related to the blood lipid concentrations. While the D/D genotype was related with increased LDL and TG level, it was related with decreased HDL concentrations in men. In our study, there was no significant difference.

Nonalcoholic fatty liver disease shows a very close association with metabolic syndrome, which also includes HT and thus with cardiovascular diseases.^26^ In our study, the NAFLD group contained four (12.9%) HT patients. The ACE level was not different in the subjects with and without HT. Again, the numbers of the subgroups with HT were similar. Angiotensin-converting enzyme inhibitors and angiotensin receptor blockers decrease the vasoconstrictive effect of AT-II and cause a decrease in the vascular resistance by decreasing the level of AT-II and preventing binding to AT-II receptor. Angiotensin-II, which is considered one of the very potent fibrogenic molecules that activate hepatic satellite cells, encourages extracellular matrix deposition, myelofibroblast proliferation and contraction, and inflammatory cytokine release in chronic liver diseases.^27^

Conclusively, we found that the ACE level and polymorphism were not directly related with the etiology and progression of NAFLD. However, new studies examining the factors including ACE sensitivity which might be increased at the molecular level in the fatty hepatic tissue and the local RAS level in the hepatic tissue are needed considering that fact that beneficial results have been obtained with ACE-blocking agents in different studies.
